# 4-Hydroxy­pyridinium hydrogen sulfate

**DOI:** 10.1107/S1600536809048533

**Published:** 2009-11-21

**Authors:** Li-Hua Huo, Ying-Ming Xu, Shan Gao, Seik Weng Ng

**Affiliations:** a College of Chemistry and Materials Science, Heilongjiang University, Harbin 150080, People’s Republic of China; b Department of Chemistry, University of Malaya, 50603 Kuala Lumpur, Malaysia

## Abstract

The crystal structure of the title salt, C_5_H_6_NO^+^·HSO_4_^−^, consists of planar(r.m.s. deviation = 0.001 Å) 4-hydroxy­pyridinium cations and hydrogen sulfate anions which are hydrogen bonded into a layer motif. In the anion, the S—O bond [1.551 (2) Å] involving the O atom bearing the acid H atom is longer than the other three S—O bonds, which range from 1.437 (1) to 1.454 (1) Å.

## Related literature

For the crystal structures of bis­(4-hydroxy­pyridinium) sulfate monohydrate and tris­(4-hydroxy­pyridinium) hydrogen disulfate monohydrate, see: Xu *et al.* (2009*a* ▶,*b* ▶).
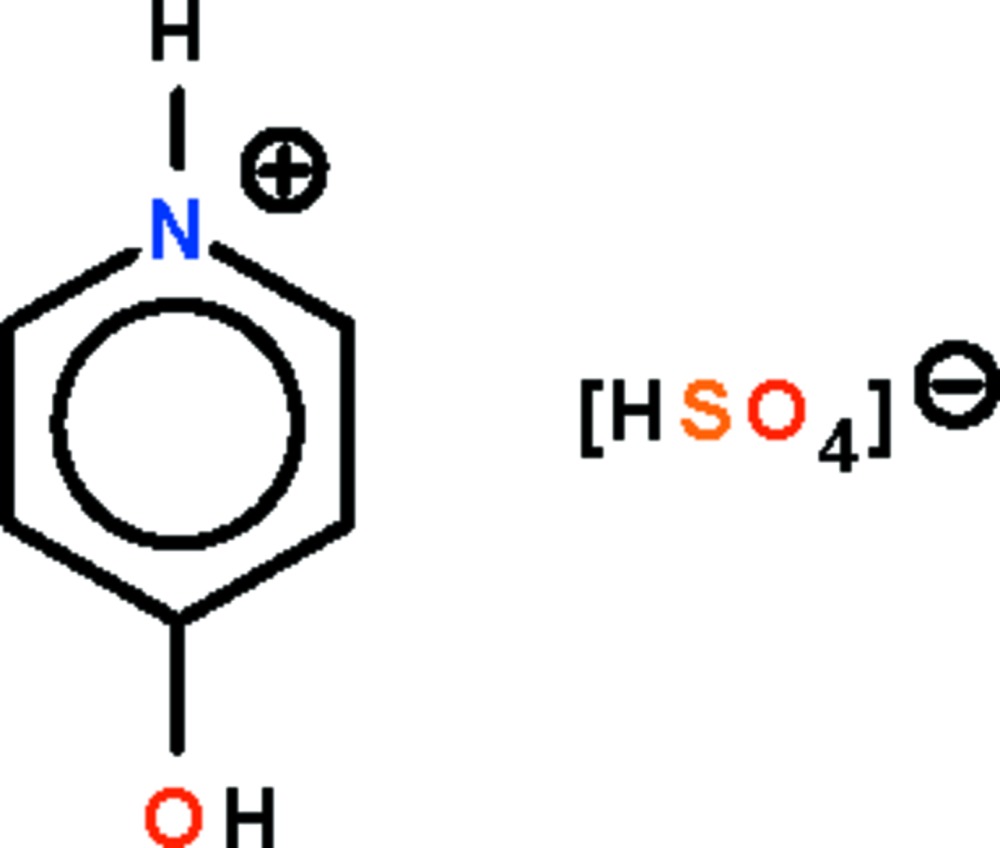


## Experimental

### 

#### Crystal data

C_5_H_6_NO^+^·HSO_4_^−^
*M* *_r_* = 193.18
Monoclinic, 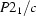

*a* = 10.4541 (7) Å
*b* = 10.7017 (6) Å
*c* = 6.8397 (4) Å
β = 96.503 (2)°
*V* = 760.28 (8) Å^3^
*Z* = 4
Mo *K*α radiation
μ = 0.41 mm^−1^
*T* = 293 K
0.27 × 0.21 × 0.15 mm

#### Data collection

Rigaku R-AXIS RAPID IP diffractometer
Absorption correction: multi-scan (*ABSCOR*; Higashi, 1995 ▶) *T*_min_ = 0.898, *T*_max_ = 0.941
7273 measured reflections
1729 independent reflections
1612 reflections with *I* > 2σ(*I*)
*R*_int_ = 0.017

#### Refinement

*R*[*F* ^2^ > 2σ(*F* ^2^)] = 0.036
*wR*(*F* ^2^) = 0.098
*S* = 1.03
1729 reflections
137 parameters
7 restraints
All H-atom parameters refined
Δρ_max_ = 0.52 e Å^−3^
Δρ_min_ = −0.39 e Å^−3^

### 

Data collection: *RAPID-AUTO* (Rigaku, 1998 ▶); cell refinement: *RAPID-AUTO*; data reduction: *CrystalClear* (Rigaku/MSC, 2002 ▶); program(s) used to solve structure: *SHELXS97* (Sheldrick, 2008 ▶); program(s) used to refine structure: *SHELXL97* (Sheldrick, 2008 ▶); molecular graphics: *X-SEED* (Barbour, 2001 ▶); software used to prepare material for publication: *publCIF* (Westrip, 2009 ▶).

## Supplementary Material

Crystal structure: contains datablocks global, I. DOI: 10.1107/S1600536809048533/xu2677sup1.cif

Structure factors: contains datablocks I. DOI: 10.1107/S1600536809048533/xu2677Isup2.hkl

Additional supplementary materials:  crystallographic information; 3D view; checkCIF report

## Figures and Tables

**Table 1:** Selected bond lengths (Å)

S1—O1	1.445 (1)
S1—O2	1.551 (2)
S1—O3	1.437 (1)
S1—O4	1.454 (1)

**Table 2:** Hydrogen-bond geometry (Å, °)

*D*—H⋯*A*	*D*—H	H⋯*A*	*D*⋯*A*	*D*—H⋯*A*
O2—H2⋯O4^i^	0.85 (1)	1.77 (1)	2.603 (2)	168 (3)
O5—H5⋯O1	0.85 (1)	1.77 (1)	2.6166 (19)	175 (3)
N1—H1⋯O3^ii^	0.84 (1)	2.04 (1)	2.8529 (19)	163 (2)

Symmetry codes: (i) 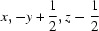; (ii) 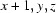.

**Fig. 1.**
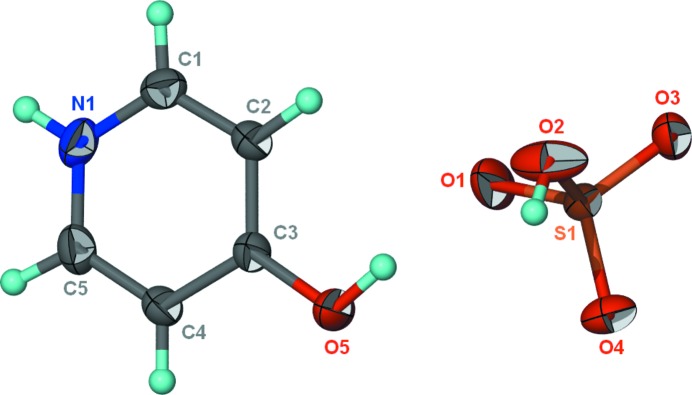
Thermal ellipsoid plot (Barbour, 2001) of [C5H6NO]+ [HSO4]- at the 50% probability level; hydrogen atoms are drawn as spheres of arbitrary radius.

## supplementary crystallographic information

### Experimental

The compound is a side product that was obtained when commercially available 4-hydroxypyridine-3-sulfonic acid was recrystallized from water. Its crystals were obtained from a water solution.

### Refinement

Carbon-bound H-atoms refined with a C–H distance restraint of 0.95±0.01 Å; their temperature factors were refined. The nitrogen- and oxygen-bound H-atoms were refined with a distance restraint of N–H = O–H = 0.85±0.01 Å; their temperature factors were refined.

### Crystal data

**Table:**

C_5_H_6_NO^+^·HSO_4_^−^	*F*(000) = 400
*M**_r_* = 193.18	*D*_x_ = 1.688 Mg m^−^^3^
Monoclinic, *P*2_1_/*c*	Mo *K*α radiation, λ = 0.71073 Å
Hall symbol: -P 2ybc	Cell parameters from 6627 reflections
*a* = 10.4541 (7) Å	θ = 3.6–27.4°
*b* = 10.7017 (6) Å	µ = 0.41 mm^−^^1^
*c* = 6.8397 (4) Å	*T* = 293 K
β = 96.503 (2)°	Prism, colorless
*V* = 760.28 (8) Å^3^	0.27 × 0.21 × 0.15 mm
*Z* = 4	

### Data collection

**Table:**

Rigaku R-AXIS RAPID IP diffractometer	1729 independent reflections
Radiation source: fine-focus sealed tube	1612 reflections with *I* > 2σ(*I*)
graphite	*R*_int_ = 0.017
ω scan	θ_max_ = 27.4°, θ_min_ = 3.6°
Absorption correction: multi-scan (*ABSCOR*; Higashi, 1995)	*h* = −13→13
*T*_min_ = 0.898, *T*_max_ = 0.941	*k* = −13→13
7273 measured reflections	*l* = −8→8

### Refinement

**Table:**

Refinement on *F*^2^	Primary atom site location: structure-invariant direct methods
Least-squares matrix: full	Secondary atom site location: difference Fourier map
*R*[*F*^2^ > 2σ(*F*^2^)] = 0.036	Hydrogen site location: inferred from neighbouring sites
*wR*(*F*^2^) = 0.098	All H-atom parameters refined
*S* = 1.03	*w* = 1/[σ^2^(*F*_o_^2^) + (0.0587*P*)^2^ + 0.3712*P*] where *P* = (*F*_o_^2^ + 2*F*_c_^2^)/3
1729 reflections	(Δ/σ)_max_ = 0.001
137 parameters	Δρ_max_ = 0.52 e Å^−^^3^
7 restraints	Δρ_min_ = −0.39 e Å^−^^3^

### Fractional atomic coordinates and isotropic or equivalent isotropic displacement parameters (Å^2^)

**Table:**

	*x*	*y*	*z*	*U*_iso_*/*U*_eq_	
S1	0.15352 (4)	0.36664 (3)	0.22053 (6)	0.02984 (15)	
O1	0.28767 (13)	0.37320 (14)	0.2986 (3)	0.0580 (4)	
O2	0.1438 (2)	0.41518 (14)	0.0056 (2)	0.0657 (5)	
O3	0.07735 (11)	0.45271 (12)	0.3196 (2)	0.0423 (3)	
O4	0.10415 (14)	0.23950 (12)	0.21136 (18)	0.0430 (3)	
O5	0.46894 (12)	0.20467 (13)	0.2977 (2)	0.0486 (4)	
N1	0.82340 (14)	0.36324 (15)	0.3509 (2)	0.0416 (4)	
C1	0.72047 (18)	0.43863 (17)	0.3364 (3)	0.0388 (4)	
C2	0.59869 (16)	0.39037 (16)	0.3180 (3)	0.0351 (4)	
C3	0.58308 (15)	0.26042 (15)	0.3148 (2)	0.0320 (3)	
C4	0.69235 (16)	0.18433 (16)	0.3312 (3)	0.0359 (4)	
C5	0.81115 (16)	0.23848 (19)	0.3484 (3)	0.0410 (4)	
H1	0.8975 (14)	0.395 (2)	0.366 (4)	0.063 (7)*	
H2	0.134 (3)	0.357 (2)	−0.078 (4)	0.079 (9)*	
H5	0.412 (2)	0.2615 (19)	0.293 (4)	0.063 (7)*	
H1A	0.735 (2)	0.5257 (10)	0.338 (3)	0.044 (6)*	
H2A	0.5283 (16)	0.4452 (18)	0.302 (3)	0.049 (6)*	
H4	0.6824 (19)	0.0967 (9)	0.330 (3)	0.040 (5)*	
H5A	0.8881 (16)	0.191 (2)	0.356 (4)	0.061 (7)*	

### Atomic displacement parameters (Å^2^)

**Table:**

	*U*^11^	*U*^22^	*U*^33^	*U*^12^	*U*^13^	*U*^23^
S1	0.0273 (2)	0.0272 (2)	0.0352 (2)	−0.00275 (13)	0.00428 (15)	−0.00138 (13)
O1	0.0264 (7)	0.0495 (9)	0.0963 (12)	0.0041 (5)	−0.0012 (7)	−0.0159 (8)
O2	0.1253 (16)	0.0347 (7)	0.0384 (7)	−0.0135 (9)	0.0154 (8)	0.0017 (6)
O3	0.0312 (6)	0.0428 (7)	0.0538 (8)	0.0005 (5)	0.0090 (5)	−0.0101 (6)
O4	0.0581 (8)	0.0317 (6)	0.0391 (6)	−0.0130 (5)	0.0045 (5)	0.0004 (5)
O5	0.0254 (6)	0.0346 (7)	0.0852 (11)	−0.0021 (5)	0.0041 (6)	−0.0049 (6)
N1	0.0289 (7)	0.0507 (9)	0.0457 (8)	−0.0105 (6)	0.0064 (6)	−0.0084 (7)
C1	0.0439 (9)	0.0337 (8)	0.0397 (9)	−0.0068 (7)	0.0086 (7)	−0.0045 (7)
C2	0.0329 (8)	0.0303 (8)	0.0424 (9)	0.0037 (6)	0.0052 (7)	−0.0022 (6)
C3	0.0268 (7)	0.0316 (8)	0.0376 (8)	−0.0003 (6)	0.0037 (6)	−0.0026 (6)
C4	0.0315 (8)	0.0308 (8)	0.0454 (9)	0.0034 (6)	0.0037 (7)	−0.0031 (7)
C5	0.0279 (8)	0.0485 (10)	0.0466 (9)	0.0043 (7)	0.0040 (7)	−0.0058 (8)

### Geometric parameters (Å, °)

**Table:**

S1—O1	1.445 (1)	N1—H1	0.841 (10)
S1—O2	1.551 (2)	C1—C2	1.366 (2)
S1—O3	1.437 (1)	C1—H1A	0.945 (10)
S1—O4	1.454 (1)	C2—C3	1.400 (2)
S1—O2	1.5514 (15)	C2—H2A	0.938 (10)
O2—H2	0.848 (10)	C3—C4	1.397 (2)
O5—C3	1.3273 (19)	C4—C5	1.363 (2)
O5—H5	0.849 (10)	C4—H4	0.943 (9)
N1—C1	1.340 (2)	C5—H5A	0.947 (10)
N1—C5	1.341 (3)		
			
O3—S1—O1	111.16 (8)	C2—C1—H1A	121.6 (13)
O3—S1—O4	114.04 (8)	C1—C2—C3	118.86 (16)
O1—S1—O4	112.73 (9)	C1—C2—H2A	119.0 (14)
O3—S1—O2	104.60 (9)	C3—C2—H2A	122.1 (14)
O1—S1—O2	106.91 (11)	O5—C3—C4	117.63 (15)
O4—S1—O2	106.71 (8)	O5—C3—C2	123.36 (15)
S1—O2—H2	113 (2)	C4—C3—C2	119.01 (15)
C3—O5—H5	107.5 (19)	C5—C4—C3	119.20 (16)
C1—N1—C5	121.59 (15)	C5—C4—H4	121.5 (12)
C1—N1—H1	119.2 (19)	C3—C4—H4	119.3 (12)
C5—N1—H1	119 (2)	N1—C5—C4	120.59 (16)
N1—C1—C2	120.75 (16)	N1—C5—H5A	116.9 (16)
N1—C1—H1A	117.6 (13)	C4—C5—H5A	122.5 (16)
			
C5—N1—C1—C2	−0.1 (3)	O5—C3—C4—C5	179.79 (17)
N1—C1—C2—C3	0.1 (3)	C2—C3—C4—C5	−0.4 (3)
C1—C2—C3—O5	179.94 (17)	C1—N1—C5—C4	−0.1 (3)
C1—C2—C3—C4	0.2 (2)	C3—C4—C5—N1	0.4 (3)

### Hydrogen-bond geometry (Å, °)

**Table:**

*D*—H···*A*	*D*—H	H···*A*	*D*···*A*	*D*—H···*A*
O2—H2···O4^i^	0.85 (1)	1.77 (1)	2.603 (2)	168 (3)
O5—H5···O1	0.85 (1)	1.77 (1)	2.6166 (19)	175 (3)
N1—H1···O3^ii^	0.84 (1)	2.04 (1)	2.8529 (19)	163 (2)

Symmetry codes: (i) *x*, −*y*+1/2, *z*−1/2; (ii) *x*+1, *y*, *z*.
